# The condensed tannins of Okoume (*Aucoumea klaineana* Pierre): A molecular structure and thermal stability study

**DOI:** 10.1038/s41598-020-58431-7

**Published:** 2020-02-04

**Authors:** Starlin Péguy Engozogho Anris, Arsène Bikoro Bi Athomo, Rodrigue Safou Tchiama, Francisco José Santiago-Medina, Thomas Cabaret, Antonio Pizzi, Bertrand Charrier

**Affiliations:** 1 CNRS/Université de Pau des Pays de l’Adour, Institut des Sciences analytiques et de physico-chimie pour l’environnement et les matériaux, Xylomat, UMR5254, 40004 Mont-de-Marsan, France; 2 Laboratoire de Recherche et de Valorisation du Matériau Bois (LaReVa Bois). Ecole Normale Supérieure d’Enseignement Technique (ENSET), BP 3989 Libreville, Gabon; 3 Laboratoire des Substances Naturelles et de Synthèse Organométalliques (LASNSOM), Unité de Recherche en Chimie, Université des Sciences et Techniques de Masuku, BP 941 Franceville, Gabon; 4 EINSTIB-LERMAB, Université de Lorraine, 27 rue Philippe Seguin, BP 1041, 88051 Epinal, France

**Keywords:** Environmental sciences, Pollution remediation

## Abstract

In order to promote convenient strategies for the valorization of *Aucoumea klaineana* Pierre (Okoume) plywood and sawmill wastes industry in the fields of adhesives and composites, the total phenolic content of Okoume bark, sapwood and heartwood was measured. The molecular structure of tannins extracted from the bark was determined by Matrix Assisted Laser Desorption/Ionization Time-Of-Flight (Maldi-ToF) mass spectrometry and Fourier transform infrared spectroscopy (FTIR). The total phenolic content displayed significant difference (p = 0.001) between the bark, sapwood and heartwood which decreased as follows: 6 ± 0.4, 2 ± 0.8 and 0.7 ± 0.1% respectively. The pro-anthocyanidins content was also significantly different (p = 0.01) among the three wood wastes, and the bark was the richest in condensed tannins (4.2 ± 0.4%) compared to the sapwood (0.5 ± 0.1%) and heartwood (0.2 ± 0.2%). Liquid chromatography coupled mass spectroscopy (LC-MS) and Maldi-ToF analysis of the bark showed for the first time that Okoume condensed tannins are fisetinidin, gallocatechin and trihydroxyflavan based monomers and complex polymers obtained with glycosylated units. No free catechin or robitinidin units were detected, whereas distinctive dihydroxy or trihydroxyflavan-3-benzoate dimers were observed in the investigated condensed tannin extracts. FTIR analysis showed the occurrence of glucan- and mannan-like sugars in the condensed tannins, and Maldi-ToF highlighted that these sugars should account for ten glycosylated units chemically bonded with two fisetinidins and one gallocatechin trimer. The condensation of these polyphenols with formaldehyde led to Stiasny numbers of 83.3, 73.3 and 53.3% for the bark, sapwood and heartwood, respectively.

## Introduction

Okoume is a tropical hardwood from central Africa and mainly located in the Republic of Congo and Gabon. That wood species represents about 130,000,000 m^3^ (∼31%) of the 400,000,000 m^3^ of Gabon forest total reserve^1^. According to Nze Nguema^2^, approximately 1,500,000 m^3^ of logs are transformed each year in Gabon according to the following distribution: sawn (825 000 m^3^), veneers (465,000 m^3^), plywood (199,500 m^3^) and trenching (10,500 m^3^). The wood wastes from the timber industry of Gabon estimated to 750,000 m^3^/year^1^ should be dominated by Okoume which account for 31% of harvested wood in Gabon. Nevertheless, the real content of unexploited Okoume bark, sapwood and heartwood wastes abandoned in forest by companies remained unknown. But a recent study which estimated the added value of unexploited forks to 120,000 FCFA/m^3^ ^3^ showed the strong financial potential of the 232, 500 m^3^ wood wastes produced per year in Gabon^2^. However, 85% of these wood wastes which are unluckily open-air burned or used as energy source for industries could receive a better attention for a suitable valorization.

In fact, the oleoresin extracted from the trunk of Okoume and used by local populations of Central Africa for several decades as a substitute for incense and for the treatment of wounds^4^ had the same medicinal properties as Okoume volatile compounds. In modern medicine, Okoume oleoresin has been used for skin, nail and hair treatment according to Delaveau and Tessier^5^, and Delaveau^6^ also mentioned some bactericide properties of that oleoresin against *Escherichia Coli*, attributed to the presence of particular phenolic compounds in Okoume essential oil. That essential oil exhibited antiradical and antioxidant activities^7^ which led to a patent of Okoume essential oils for cosmetic, pharmaceutical and dermatologic applications^8^. Moreover, the works of Rhourri‐Frih *et al*.^9^ revealed a high content of α and β-amyrin in Okoume essential oil.

Regarding Okoume sapwood and heartwood waste, many researchers proposed alternatives to bring added value on these lignocellulosic materials as composites. Therefore, Bakraji *et al*.^10^ proposed an Okoume/polyacrylamide composite with high tensile strength. Other authors investigated the interfacial bonding between the hydroxyl groups of some tropical hardwoods sawdust and succinic anhydride molecules. They found that Okoume heartwood, holocellulose and cellulose chains were weakly esterified by succinic anhydride^11,12^. This hardwood cellulose displayed short fibers which tend to aggregate in organic solutions, showing the capability of Okoume heartwood waste to be used as cellulose esters with good micromechanic and microcrystalline stability^12^. Recent studies exhibiting significant differences between Okoume sapwood and heartwood facing enzymatic hydrolysis by *Trichoderma reesei* revealed the potential of that hardwood waste to be used in biorefinery. However, the differences observed between Okoume sapwood and heartwood should be controlled by their extractives nature and composition^13^.

Despite the abundant literature cited above, the phenolic extracts content and the condensed tannin structure of Okoume have received little attention so far except the work of Mounguengui *et al*.^14^, who investigated the phenolic content of Okoume heartwood.

The aim of this study was to investigate the phenolic content of the bark, sapwood and heartwood of Okoume while the molecular structure of the bark condensed tannin extracts was studied by Maldi-ToF, LC-MS and FTIR. An attempt was made for the utilization of condensed tannins in adhesive formulations.

## Materials and Methods

### Samples

The bark, sapwood, and heartwood were collected from two Okoume trees of approximately 80 years of age. The wood was harvested at Nzamaligue in the West of Gabon by the SED (Société Equatoriale de Déroulage) in February 2016. For each tree, we collected samples on a disk of 10 cm in thickness and 85 cm in diameter. The fresh samples were placed in sterilized bags protected from light, air-dried for one week in an air-conditioned laboratory, and oven-dried (105 °C) for 48 h. The dried samples were ground so as to pass through a 180-type mesh (≈1 mm diameter) with a rotating knife grinder (Retsch SK1).

All the chemicals used in this study were purchased from Fisher Scientific and Sigma Aldrich. Solvents and reactants were used without further purification.

### Extraction of polyphenols at room temperature

350 mg of dried wood powder (M_0_) from bark, sapwood and heartwood samples were mixed separately at room temperature with 30 ml of acetone/water solution (7:3, v/v). The mixtures were stirred for 3 h. The supernatant was recovered and the acetone was evaporated. Each extraction was repeated four times per each tissue type (bark, sapwood and heartwood) collected from each tree.

### Total phenolic content measurement

The total phenol content was determined by the Folin-Ciocalteu method^15^ as follows: 1 ml of bark, sapwood and heartwood extracts were diluted with 9 ml of distilled water, and 0.5 ml of the diluted extracts was poured into 2 ml of Folin-Ciocalteu reagent (1/10, v/v in distilled water). Then, 2.5 ml of sodium carbonate solution (0.7 M) was added to the Folin-Ciocalteu reagent containing the extracts. The final solution obtained was placed into test tubes and left for 5 min in a water bath maintained at 50 °C. The absorbance was registered at 760 nm. The results were expressed as the gallic acid equivalent (GAE), based on the extracted dry powder amount. The total phenol content was determined according to the following Eq. 1:

1 $$Total\,phenol\,( \% )=\frac{C\times D\times V}{1000\times {M}_{0}}\times 100$$

C: Total phenol concentration (ppm). D: degree of dilution (10). V: volume of starting solution (30 ml). M_0_: mass of dry sawdust (mg).

### Determination of proanthocyanidin content

#### Anthocyans measurement by the acid hydrolysis in butanol method

5 ml of ferrous sulfate solution obtained by dissolving 77 mg of FeSO_4_·7H_2_O previously poured into 500 ml of HCl (37%) in nBuOH (2/3, v/v) were added to 0.5 ml of dried aqueous extracts from bark, sapwood and heartwood extracts. The mixtures obtained were placed for 15 min in a water bath maintained at 95 °C. After cooling, the absorbances were recorded at 530 nm and the results were expressed as cyanidin equivalent based on the dried wood extracts content. Proanthocyanidin (PA) was determined^16^ according to the following Eq. 2:

2 $$[PA]=\frac{A\times V\times D\times V^{\prime} \times M}{{\rm{\varepsilon }}\times v\times m}\times 100$$

PA: Proanthocyanidins content (mg cyanidin Equivalent/g dry weight expressed as mg cyaE/g bark); V: volume of reaction (ml). D: dilution factors (10). V′: volume of the aqueous extract recovered after extraction with diethyl ether (ml). M: cyanidin molar mass (287 g/mol). v: volume of the sample (ml). A: absorbance of the sample. m: mass of dry sawdust samples (g). ε: molar extinction coefficient (34700 M^−1^cm^−1^) according to Scalbert *et al*.^17^.

#### Condensed tannins measurement by the acid condensation of vanillin method

Condensed tannins in an acid medium were measured according to the vanillin condensation method described by Broadhurst *et al*.^18^ as follows: 0.5 ml of aqueous extracts from bark, sapwood and heartwood contained in a tube were mixed with 3 ml of vanillin reagent dissolved in methanol (4%, w/v). Then, 1.5 ml of concentrated HCl (37%) was added, and the mixture was kept in the dark at 20 °C for 15 min. Absorbances were registered at 500 nm. The results obtained were expressed as catechin equivalence (CE) based on the amount of dry extracted samples. The calibration was carried out using an aqueous solution of catechin (30 mg/l). However, the standard solutions contained 0, 15, 25, 35, 45, 55, 65, 75, 85, 95 and 100 ppm of catechin.

#### Extraction of tannins for thermal and stiasny number analysis

Tannin extraction from Okoume bark was carried out in water containing 5% of sodium hydroxide, 0.25% of sodium sulfite and 0.25% of sodium bisulfite, with a sample-to-water ratio equal to 1/9. The bark sawdust was immersed in water maintained under magnetic stirring for 3 h at 80 °C. The tannin extracts obtained were filtered and oven-dried at 50 °C.

#### Stiasny number determination

The reactivity of extracts with formaldehyde was determined by measuring the Stiasny number (SI) as described by Voulgaridis *et al*.^19^. A solution of extract of concentration 4 g/l was prepared. 25 ml of this solution was put in round bottom flask and 5 ml of formaldehyde 37% and 2.5 ml of HCl 10 M were added. The mixture was heated under reflux for 30 min. The residue was filtered through sintered glass n°2 or 4. The precipitate was washed with water and dried at 105 °C until constant weight. The reactivity was calculated with the formula below (3):

3 $${\rm{SI}}=\frac{A}{B}\times 100$$

With SI: Stiasny number. *A*: dry weight of precipitate (mg). *B*: dry weight of extracts (mg).

#### LC-MS analysis

350 mg of dried wood powder from bark samples were mixed separately at room temperature with 30 ml of methanol/water solution (ratio 4:1, v/v). After 3 h of stirring, the supernatant was recovered, then analyzed via LC-DAD (Ultimate 3000, Thermo Scientific), using an Acclaim Polar Advantage II (Thermo Scientific) in methanol (A)/water (B) gradient mode at 1 ml/min (injection volume 10 μl). The gradient elution program was set as follows: 0–9 min. (95% B), 9–16 min. (75% B), 16–25 min. (60% B), 25–35 min. (50% B), 35–52 min. (0.0% B), 52–62 min. (95% B). Then, the elution gradient was linearly ramped down to 60% A for 2 min and so maintained for 9 min to condition the column for the next injection. The column was connected to an electrospray hybrid linear ion Orbitap mass analyzer (LTQ Orbitrap Velos, Thermo-Fisher, Bremen, Germany). The electrospray spray voltage was 3.8 kV. The LC-MS analysis was used in negative ion detection mode. Data were analyzed with the Xcalibur software.

#### Maldi-ToF of bark analysis

The acetone/water tannin extracts from Okoume bark were oven-dried at 105 °C for 24 h and dissolved in an acetone/water (1:1, v/v) solution up to 7.5 mg/ml. To increase ion formation, 1.5 μl of NaCl solution (0.1 M) in a methanol/water mixture (1:1, v/v**)** was added and placed on the Maldi target. The sample solution and the matrix were then mixed in equal amounts, and 1.5 μl of the resulting solution was placed on the Maldi target. A matrix of 2,5-dihydroxy benzoic acid was used. Red phosphorous (500–3000 Da) was used as reference for spectrum calibration. Finally, after evaporation of the solvent, the Maldi target was introduced into the spectrometer. The spectra were recorded on a KRATOS AXIMA Performance mass spectrometer from Shimadzu Biotech (Kratos Analytical Shimadzu Europe Ltd., Manchester, UK). The irradiation source was a pulsed nitrogen laser with a wavelength of 337 nm. The length of one laser pulse was 3 ns. Measurements were carried out using the following conditions: polarity-positive, flight path linear, 20 kV acceleration voltages, 100–150 pulses per spectrum. The delayed extraction method was used, applying delay times of 200–800 ns. The software Maldi-MS was used for the data analysis.

#### Fourier transform infrared spectroscopy (FTIR)

The acetone/water tannins extracted from Okoume bark were characterized by FTIR analysis. The samples were oven-dried at 105 °C for 24 h prior to any analysis, 5 mg of dried tannins powder were placed in the crystal device, and the contact was obtained by applying a force of 150 N on the sample. 32 scans were used with a resolution of 4 cm^−1^ in the range 4000–600 cm^−1^. All the FTIR assays were carried out in ATR (attenuated total reflection) single bounce mode with a Perkin Elmer Frontier spectrophotometer equipped with a diamond/ZnSe crystal for tannin analysis. The spectra were collected and analyzed using Spectrum software (Perkin Elmer).

#### Thermogravimetric analysis (TGA analysis)

Thermal decomposition was performed using a TGA Q50 from TA Instruments. The temperature program was from 25 to 600 °C at a heating rate of 10 °C/min. the measurement were conducted in a nitrogen atmosphere (40 ml/min). The results were analyzed with TA Instruments’ Universal Analysis 2000 software.

#### Differential scanning calorimetry (DSC)

The differences in heat exchange between an analysis sample and a reference were measured on a DSC Q20 (TA instruments) equipped with a rapid cooling system. Samples of Okoume tannins were weighed (≈7 mg) in standard aluminium pans (TA Instruments) and data acquisition was carried out using the Universal Analysis 2000 program (TA Instruments). The measurements were performed in nitrogen (40 ml/min) with a standard heating rate of 10 °C/min from room temperature to 400 °C.

## Results and Discussion

### Total phenolic content

The amount of phenolic compounds obtained from Okoume was shown in Table 1. The results reported as mathematical mean +/− SD of the technical replicates showed differences between the phenolic content of the bark, sapwood and heartwood. The bark of the studied samples was the richest in phenolic compounds (6 ± 0.35% of dry wood) while the heartwood was the least abundant (0.74 ± 0.35% of dry wood). Nevertheless, the phenolic content found in the heartwood was close to that published by Mounguengui *et al*.^14^, who obtained 0.64 ± 0.05% (of dry wood) of phenolic compounds in Okoume heartwood. Furthermore, the Okoume bark phenolic content was higher than that found in the bark of African mahogany (*Khaya ivorensis*) (1.54 ± 0.39% of dry wood) treated in the same experimental conditions^20^, sapwoods phenolic content was not different, and the heartwood phenolic content was higher in African mahogany’s (1.79 ± 0.11% of dry wood) than in Okoume.

**Table 1:** Total phenolic, anthocyan (vanillin assay) and pro-anthocyanidin (ButOH-HCl assay) content expressed in % of dry weight and Stiasny number of Okoume expressed in %.

Okoume type sample	Total phenolic content (% of dry weight)*	Vanillin assay (% of dry weight)*	ButOH-HCl assay (% of dry weight)*	Stiasny number (%)*
Bark	6 ± 0.4	4.2 ± 0.4	0.6 ± 0.1	83.3 ± 11.6
Sapwood	2 ± 0.8	0.5 ± 0.1	0.3 ± 0.04	73.3 ± 15.3
Heartwood	0.7 ± 0.1	0.2 ± 0.2	0.1 ± 0.2	53.3 ± 11.6

N = 8, represents the total number of technical replicates extraction per sample type (bark, sapwood, heartwood) obtained from the two logs.

*Means ± SD.

### Condensed tannins content

#### Catechin and proanthocyanidins equivalent content

The results obtained from the vanillin assay and reported as mathematical mean +/− SD of the technical replicates are listed in Table 1. They exhibited differences between the condensed tannins content bearing a hydroxyl group at the “meta” position of the flavanol unit of the bark, sapwood and heartwood of Okoume. That result which highlighted a high content of anthocyan in the bark was corroborated by those obtained from the ButOH-HCl assay indicating the high content of Okoumes’s bark in pro-athocyanidin type condensed tannins. Nevertheless, the dominating condensed tannins content in Okoume bark is in agreement with that ascertained by Stevanovic and Perrin^21^, who defined tannins as polyphenolic compounds present in plants, and the bark of trees may have the highest amount. Moreover, this study has shown the strong condensed tannins content of Okoume bark (40.19 ± 3.6 mgCE/g of dry bark) compared to the bark of *Pinus pinaster* (2.18 ± 0.57 mgCE/g of dry)^22^ which is used as raw material for commercial tannins.

### LC-MS, Maldi-ToF and FTIR analysis

**Figure 1:**
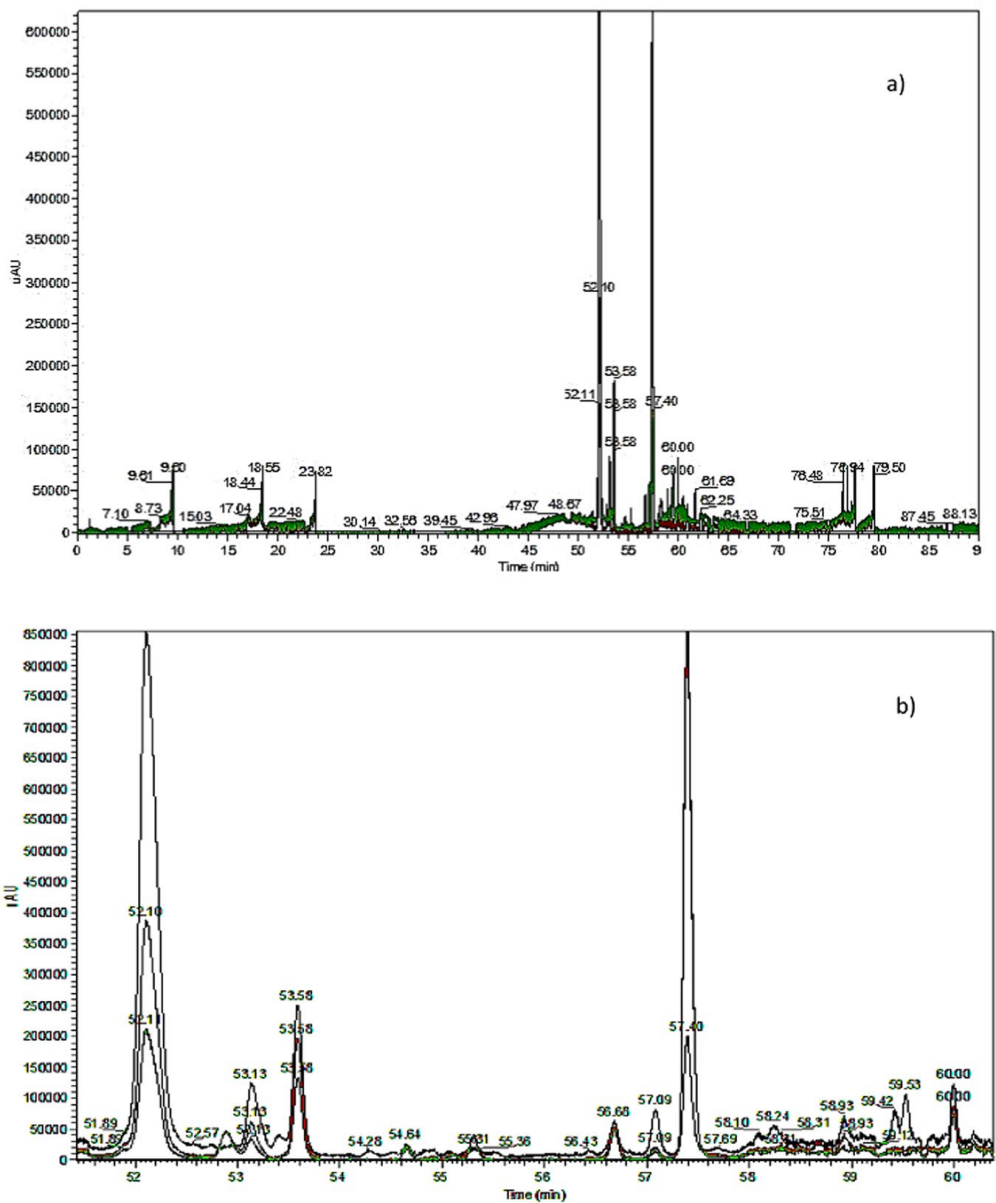
LC-MS spectra of Okoume bark tannins presented at three different overlaying wavelengths (220 = black, 254 = red and 280 nm = green. Detected with a Diode Array Detector (DAD) at retention time (a = 0–90 mm, b = 50–60 mm).

**Figure 2:**
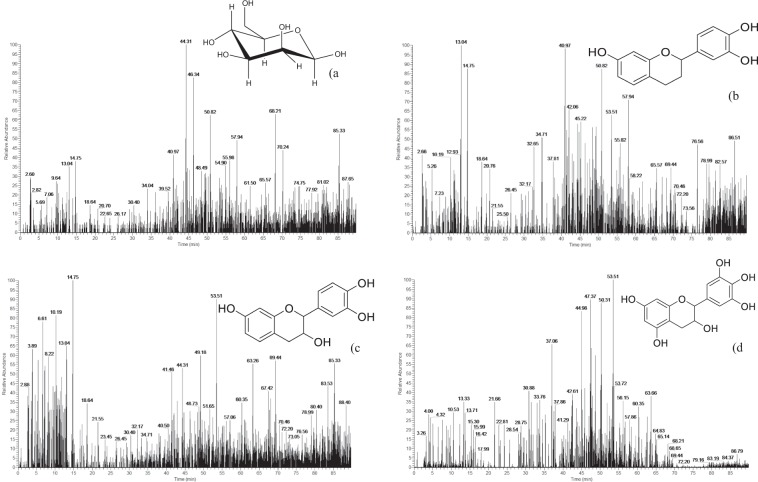
Mass fragments of the main monomers (a = glucose, b = trihydroxyflavan-3-ol, c = fisetinidin, d = gallocatechin).

LC-MS is an analytical method to identify and quantify phenolic compounds in plants^23–25^. It was used here to characterize the phenolic moieties including flavonoid structures of Okoume extracts. The ionization of oligomer structures led to mass fragments of main monomers present in Okoume condensed tannins which appeared in the ionized [M-H]^−^ form. The results obtained with an output predominance around 40–60 and 60–85 minutes were collected (Figs. 1–2) and compared to those displayed by Maldi-Tof analysis for a further identification of major monomers or oligomers from Okoume extracts. The molecules were identified using MS spectra.

#### Sugar analysis of tannin extracts

**Figure 3:**
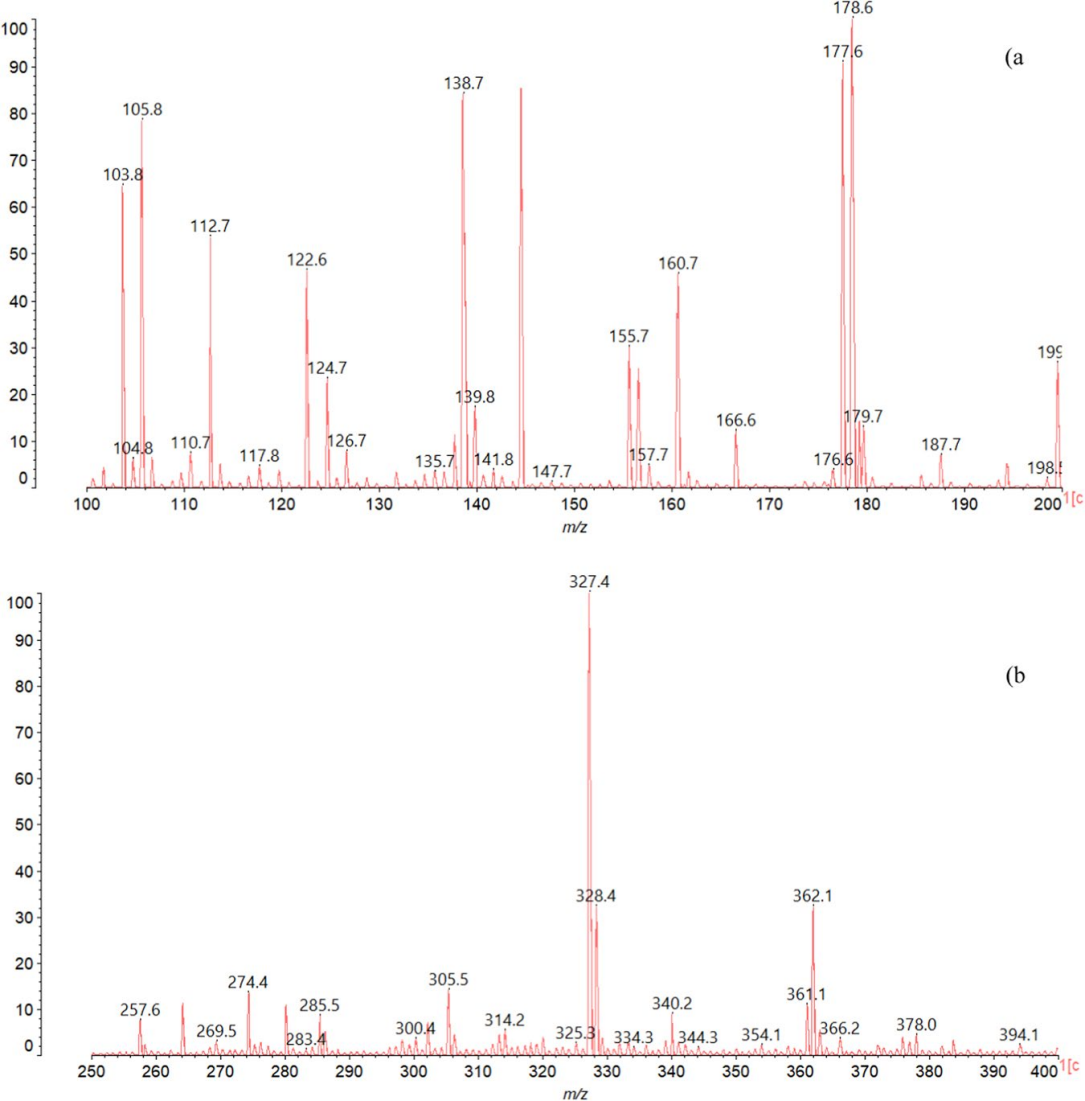
Maldi-ToF spectrum of Okoume bark tannin extracts in the ranges 100–200 Da (**a**) and 250–400 Da (**b**).

The sugars released in the condensed tannin bark extracts were studied in the range 100–200 Da of Maldi-ToF spectrum (Fig. 3a). It was noteworthy that in Okoume bark the peak at 126.7 Da relates to a cleavage mechanisms involving the glucose ring as previously found by Ricci *et al*.^26^ in the fragmentation pattern related to polygalloylglucose structures. The presence of glucose or other hexose units should be corroborated by the strong peak at 178.6 Da and the series of peaks at 177.6 and 179.7 Da from glucose or other hexose degradation^11,13,20^. The presence of glucose in Okoume bark extracts was supported by LC-MS results (Fig. 2a) which exhibited masses fragments at 179.9/180 Da assigned to glucose monomer^16^. However, the peaks at 177.6 and 179.7 Da (Fig. 3a) assigned to glucose which has lost 2 × 1 H during the Maldi-ToF process^20^ should also be attributed to galactose or mannose for *m/z* = 180, suggesting the occurrence of various sugar units in Okoume bark condensed tannins. Although glucose accounted for the most abundant neutral sugars of Okoume wood^11,12^; recent studies showed the presence of strong proportion of water soluble pectin structures like galactose and mannose among the neutral sugars of steam-exploded Okoume sapwood. Hence, the occurrence of other sugars than glucose in the acetone/water condensed tannins of the bark cannot completely be ruled out.

In addition, the possible mixing of glucose, galactose and mannose as oligosaccharides in Okoume condensed tannins bark was supported by FTIR spectroscopy. A strong signal at 975.77 cm^−1^ assigned to glycosidic linkage of mannans^27,28^ and C–O stretching (ν_C–O_) of galactose^28^ as well as the one at 880.7 cm^−1^ ascribed to H in the equatorial direction at position 2 of acetylated mannose^28^ was consistent with the presence of mannose and galactose units in those condensed tannins. On the other hand, the sharp peak at 1111.6 cm^−1^ assigned to C–OH deformation of β(1 → 3)glucanes^27^, consistent with the presence of glucose units in Okoume condensed tannins, could alternatively be assigned to ν_C–O_ of exocyclic galactosyl units ring appearing at 1112 cm^−1^ ^29^, suggesting a potential presence of galactose among the sugars bonded to the condensed tannins extracted from Okoume bark. Therefore, the shoulder at 781.1 cm^−1^ assigned to β-D-glycopyrannoside structure as stated by Kato *et al*.^28^ indicated that oligosaccharide chains should be linked to the condensed tannins extracted from Okoume bark. The peak of weak intensity at 135.7 Da resulting from deoxyribose moiety^20^ showed a low content of that sugar in the condensed tannins extracted from Okoume bark (Fig. 3a).

#### Polyphenols analysis of acetone/water extracts

The acetone/water extracts of Okoume bark is redolent of typical structures of condensed tannins as shown in the spectrum collected in Fig. 3b and the corresponding indexations listed in Tables 2 and 3. Signals in the range 250–400 Da shown a peak at 274.4 Da previously assigned to fisetinidin (A) in other wood or lignocellulosic materials^30,31^ while gallocatechin monomer (D) appeared at 305.5 Da. The presence of these compounds agreed with these obtained by LC-MS where they appear in ionized form [M-H]^−^ of *m/z* = 272 and 304 Da for fisetinidin and gallocatechin, respectively^32,33^ (Fig. 2). However, catechin/robitinidin monomer was not found at *m/z* = 290 Da in the Maldi-ToF spectra of Fig. 3b. These results are in agreement with the presence of condensed tannins in Okoume bark as shown by the vanillin acid method. In addition, signal at 257.6 Da assigned to trihydroxyflavan-3-ol (F) in other tannins and accounting for monomers released by LC-MS at [M–H] = 257 Da^34^ supported that the acetone/water extracts of the bark are made up of fisetinidin, gallocatechin and trihydroxyflavan-3-ol condensed tannin monomers (Fig. 2).

**Table 2:** Distribution of polyflavonoid oligomers of acetone/water condensed tannin extract of Okoume bark by Maldi-ToF.

Experimental *m/z* (Da)	Calculated *m/z* (Da)	Type unit	Oligomer type
166.6	170.10	E(gallic acid)	Monomer
179.97	180.15	Gly(Glycosyl unit)	Monomer
257.6	258.3	F_1_ (trihydroxyflavan)	Monomer
274.4	274.3	A_1_ (Fisetinidin)	Monomer
305.5	306.3	D_1_ (Gallocatechin)	Monomer
340.2	342.2	Gly_2_ (Cellobiose)	Dimer
362.1	362.0	G_1_H_1_ (Dihydroxyflavan-3-p-hydroxybenzoate) or F_1_I_1_ (Trihydroxyflavan-3-benzoate)	Dimer
405.8	404.0	A_1_Gly_1_	Dimer
422.0	420.0	F_1_Gly_1_	Dimer
438.0	436.0	Q_1_E_1_ (Epicatechin-3-gallate)	Dimer
452	452	A_1_Gly_1_	Dimer
481.6	480.0	P_2_ (Dihydroxyflavan)	Dimer
533.6	530	F_1_A_1_	Dimer
575.2	574.6	A_1_D_1_	Dimer
617	616.38	R_1_E_1_ (Isoquercetin-gallate)	Dimer
850.7	850.9	A_2_D_1_	Trimer
	851.9	P_1_D_2_	Trimer
1012.6	1012	A_2_DGly_1_	Tetramer
1174.4	1174	A_2_DGly_2_	Tetramer
1336.2	1336	A_2_DGly_3_	Pentamer
1498.1	1498	A_2_DGly_4_	Hexamer
1660.1	1660	A_2_DGly_5_	Heptamer
1822.2	1822	A_2_DGly_6_	Octamer
1984.4	1984	A_2_DGly_7_	Nonamer
2146.6	2146	A_2_DGly_8_	Decamer
2309.0	2308	A_2_DGly_9_	Undecamer
2471.0	2470	A_2_DGly_10_	Dodecamer

**Table 3:** Thermal stability estimators for Okoume bark tannins by TGA (nitrogen 60 ml.min^−1^, heating rate 10 °C.min^−1^) and DSC (nitrogen 60 ml.min^−1^, heating rate 10 °C.min^−1^).

Okoume type condensed tannins sample	TGA					DTG
	Temperature at 95 (T_95%_) and 80 (T_80%_) residual weigh		Onset temperature of first (T_d1_) and second (T_d2_) degradation		Residual weigh at 600 °C (W _600 °C_)	Maximum temperatures of degradations (T_max_)
	T_95%_	T_80%_	T_d1_	T_d2_	W_600 °C_	T_max_
Bark	101.8	420.8	81.4	220	78.5	243.99
Sapwood	101.8	358.9	81.4	220	77.1	243.99
Heartwood	100.1	276.4	83.1	220	72.7	255.64

**Figure 4:**
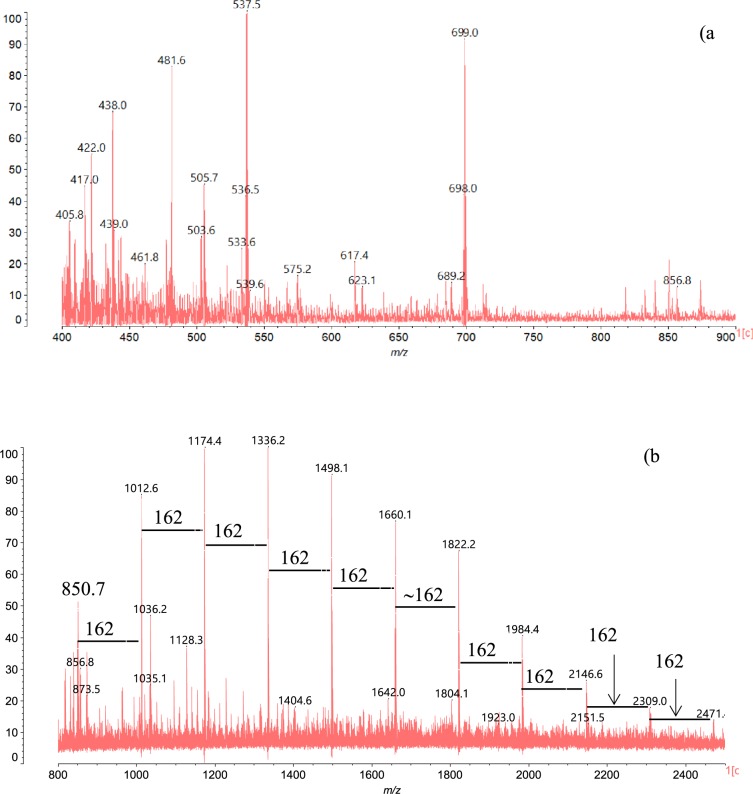
Maldi-ToF spectrum of Okoume in the range 400–900 Da (**a**) and 800–2400 Da (**b**).

The first condensed tannins dimer appeared at the 481.6 Da peak; that strong signal was assigned to 2*x*dihydroxyflavan units condensed in C_4_-C_8_ labeled P_2_ as shown in Tables 2 and 3. Another dimer was obtained at 533.6 Da for this peak was attributed to a trihydroxyflavan/fisetinidin dimer labeled F_1_A_1_ in Table 2. In addition, Maldi-ToF spectra of Fig. 4a showed a peak at 575.2 Da assigned to a fisetinidin/gallocatechin (A_1_D_1_) dimer with a loss of 3 × H as suggested in Supplementary Table 1.

**Figure 5:**
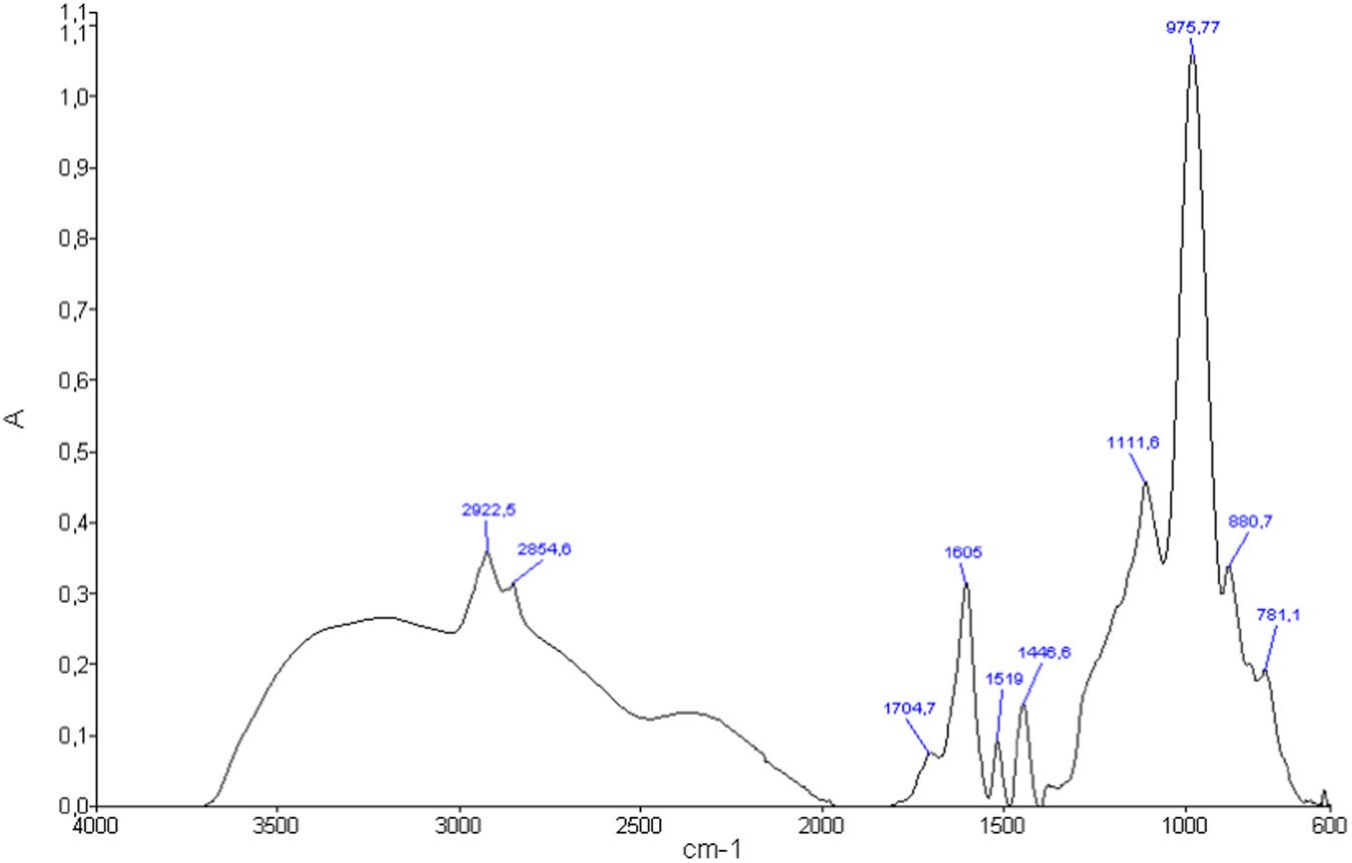
FTIR spectra of Okoume bark tannins.

The acetone/water extracts of the bark exhibited a signal at 850.7 Da assigned to a condensed tannins trimer. That peak should derive from a combination of dihydroxyflavan and two gallocatechins (P_1_D_2_, *m/z* = 851.9 Da) or from the condensation of two fisetinidins coupled with one gallocatechin (A_2_D_1_, *m/z* = 850.9 Da) as proposed in Table 2. Moreover, the presence of condensed tannins in the acetone/water extracts of the bark was corroborated by their typical aromatic C=C– stretching bands at 1605, 1519 and 1446.6 cm^−1^ ^35,36^ displayed by FTIR (Fig. 5).

However, other dimers including ester moieties were found in the acetone/water extracts. The one arising at 362.1 Da was assigned to dihydroxyflavan-3-p-hydroxybenzoate (G_1_H_1_) or to trihydroxyflavan-3-benzoate (F_1_I_1_). These compounds, previously described for *Pinus brutia* by Ucar *et al*.^37^, suggest the presence of hydrolysable tannins in the acetone/water extracts of Okoume bark. Therefore, the peak at 166.6 Da of gallic acid (E)^26^ depicted in Fig. 3a as well as the strong signal at 438.0 Da assigned to protonated epicatechin-3-gallate (Q_1_E_1_) based on previous assignment^26^, and the one at 461.8 Da (438.0 + 23 Da) ascribed to epicatechin-3-gallate sodium adducts (Q_1_E_1_, Na^+^) in grape skin^26^ should be considered as markers for galloylated units in the bark of Okoume as stated earlier for grape seed extracts^26^ (Fig. 4a). It was noteworthy that the peak at 617.4 Da in the Maldi-ToF spectrum of Okoume (Fig. 4a) suggested the presence of isoquercetin-gallate (R_1_E_1_) dimer in the investigated acetone/water extracts. The existence of hydrolysable tannins bearing carbonyl groups (C=O) in these extracts as ascertained by the discussion above was in close agreement with the LC-MS analysis. That method showed evidence of gallic acid moiety of [M–H]^−^ = 169/170 Da^38–40^ in the bark extracts of the studied hardwood species indeed; that result was in close agreement with FTIR which exhibited a specific C=O stretching shoulder of hydrolysable tannins at 1704.7 cm^−1^ ^35,36,41,42^.

Maldi-ToF analysis displayed a 340.2 Da peak previously assigned to maltose, saccharose or lactose by Sanchez-De Melo^20^, this signal supported the presence of glycosyl type dimers in the acetone/water extracts of Okoume bark. Although the neutral monosides composing saccharose (glucose) or lactose (galactose) dimers were found in Okoume whereas fructose was absent, the strong domination of glucose suggested that the peak at 340.2 Da should rather be assigned to cellobiose type dimer as previously found in *Picea abies*^43^. A series of peaks at 405.8 Da, 422.0 Da and 438.0 Da (Fig. 4a) progressively increased in steps of 16 Da starting from the peak at 405.8 Da ascribed to one dihydroxyflavan coupled with a glycosyl unit (P_1_Gly_1_) dimer of calculated *m/z* = 404 Da (Table 2). But, the dimer at 405.8 Da could also result from the loss of *2* × 16 Da by a glycosyl-3-fisetinidin (A_1_Gly_1_) dimer as suggested in Table 1 (Supplementary Table 1). Similar peak of *m/z* = 406.4 Da assigned to astringin in *Picea abies* water extracts^43^ indicated the possible existence of that stilbene glucoside in acetone/water extracts of Okoume bark. That hypothesis agreed with the traditional utilization of the bark in its endemic area (central African countries like Gabon) against diarrhea and hemorrhages^44,45^. Therefore, the peak at 422.0 Da of that glycoside series should also be assigned to a diprotonated isorhapontin, an over stilbene glucoside of *m/z* = 420.4 Da previously identified on *Picea abies* water extracts by Bianchi *et al*.^43^.

However, the peak at 438 Da that was assigned above to protonated epicatechin-3-gallate (Q_1_E_1_) and increasing for 16 Da from the peak at 422 Da should also correspond to a trimer labeled A_1_Gly_1_ deriving from a fisetinidin/glycoside dimer of calculated *m/z* = 452 Da that lost one hydroxyl group (452 Da-1 × 16 Da) during Maldi-ToF treatment as shown in Table 1 (Supplementary Table 1). Similar structures containing a carbohydrate residue linked to the C_3_ site of flavonoids have been discussed by various authors^46–48^. Ricci *et al*.^26^ assign the 461.8 Da peak preferably to epicatechin-3-gallate, but also suggest quercetin-3-glycoside (isoquercetin), considering the strong presence of glycosylated condenses tannins in the acetone/water extracts of Okoume bark.

**Figure 6:**
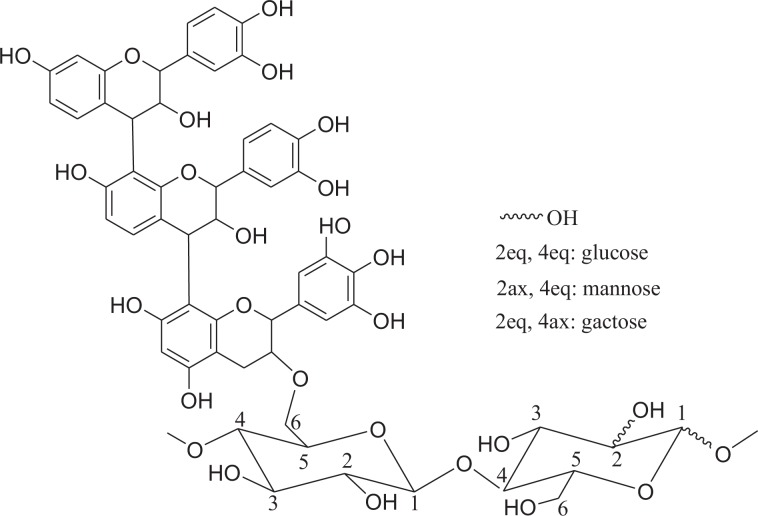
Condensed tannins linked to glycoside structures. A_2_D_1_Gly_*n*_, with 1 ≤ *n* ≤10 and Gly: glucose, mannose or galactose unit.

A fine analysis of the Maldi-ToF spectrum showed a peak series at 850.7 Da, 1012.6 Da, 1174.4 Da, 1336.2 Da, 1498.1 Da, 1660.1 Da, 1822.2 Da, 1984.4 Da, 2146.6 Da, 2309.0 Da and 2471.0 Da which increased for one typical repeating sugar unit of the 162 Da period (Fig. 4b). A similar 162 Da repetition was previously observed in water extracts of *Picea abies*, where it was assigned to sucrose-, raffinose- and stachyose-like oligosaccharides linked to condensed tannins^43^. Nevertheless, the occurrence of carbohydrates attached to condensed tannins extracted from the bark of tropical hardwood such as *Myrotharrmus flabellifofia* or *Parkia biglobosa* was supported by Pizzi and Cameron^49^, and Drovou *et al*.^48^, respectively. Therefore, the increase of 162 Da starting from the trimer at 850.7 Da consisting of two fisetinidins and one gallocatechin labeled A_2_D_1_ highlighted the existence of glycosylated oligomers including one to ten sugar units chemically bonded to the A_2_D_1_ trimer in Okoume bark acetone/water extracts. These condensed tannins/glycoside structures led to the various oligomers listed in Table 2 and designated as A_2_D_1_Gly_*n*_ (1 ≤ *n* ≤ 10), as proposed in Fig. 6.

Finally, FTIR spectroscopy showed a broad signal between 3500 and 3000 cm^−1^ (Fig. 5) attributed to O–H stretching vibration (ν_O–H_) of phenols^35,36,42^. The occurrence of these compounds in the investigated extracts was supported by the shoulder at 781.1 cm^−1^, also assigned to O–H deformation (δ_O–H_) of phenols^36^. In addition, the bands at 2922.5 and 2854.6 cm^−1^ assigned to C–H stretching (ν_C–H_) of alkyl groups^35,42,50,51^ should arise from flavonoids and sugar units identified in the acetone/water extracts of Okoume bark (Table 3).

### Stiasny number

The Stiasny number (SI) values collected in Table 1 ranged between 83.3 ± 11.6 <SI< 53.3 ± 11.6. The ANOVA test did not point out a significant difference (p > 0.05) between the SI of bark vs sapwood, nor between the SI of sapwood vs heartwood, but the difference of SI was notable between bark vs heartwood (p = 0.03). It is noteworthy that all the SI values of this study were above 46%, considered as a suitable level to produce good-quality adhesives^52^. But the SI of the bark (83%) and the sapwood (73%) were above 65%, considered to be the minimum SI value for adhesives of high quality^53^. This result showed clearly the potential of Okoume wood waste for use as raw materials high-quality adhesives. Furthermore, the average SI (83.33 ± 11.6%) in Okoume bark which had the highest condensed tannins content of the examined waste was in the same range as that found for mimosa tannins 92.2%^54^, and largely higher than that published for *Pinus pinaster* bark extracted by three different methods (17.92, 48.97 and 54.98%)^22^. That finding highlighted the strong potential of Okoume bark for use as raw material for commercial condensed tannins.

### Thermal stability control of condensed tannins

**Figure 7:**
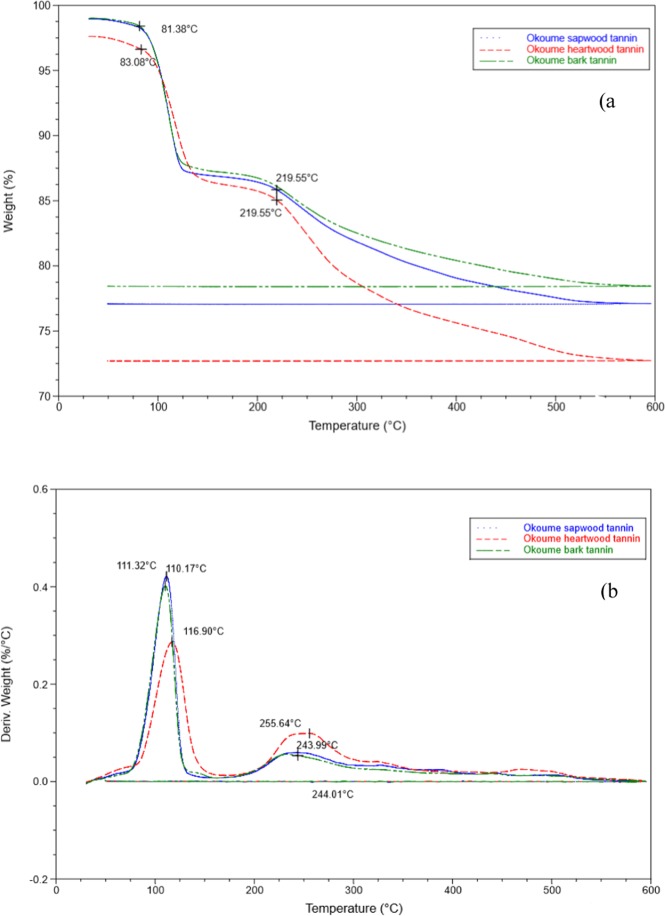
TGA (**a**) and DTG (**b**) curves of Okoume tannins heated at 10 °C/min in nitrogen, with bark tannins in green; sapwood tannins in blue and heartwood tannins in red.

The thermograms of Okoume condensed tannins obtained in nitrogen as depicted in Fig. 7 showed two major steps of tannin degradation. The first step occurred at 110.1; 111.3 and 116.9 °C in TGA analysis of the bark, sapwood and heartwood respectively. These first maximum temperatures of degradations (*T*_*max*_) should be associated to residual water removal^55^ or some easily degraded small molecular materials such as simple sugars and organic acids^56^ observed in particular in LC-MS and Maldi-ToF of acetone/water extracts of the bark (Fig. 1, Tables 2, 3). In the second step, DTG thermograms of Fig. 7b denoted *T*_*max*_ = 243.9 °C for the bark, sapwood and 255.6 °C for the heartwood condensed tannins. These second temperatures should correspond to tannins decomposition as previously observed for various condensed tannins^56–58^.

The TG analysis of Okoume condensed tannins at T < 100 °C showed that the heartwood condensed tannins underwent a mass loss of 4% for a first onset temperature of degradation or water loss of T_d1_≈83.1 °C. That temperature was slightly higher than that found for the bark and sapwood tannins (T_d1_≈81.4 °C),which underwent only slight mass loss, at 1% (Fig. 7a). The high temperature and mass loss presented by Okoume heartwood tannins should indicate at least the occurrence of high residual water within the condensed tannins of Okoume inner wood. Nevertheless, the three condensed tannins did not show difference in their temperature of degradation at 5% mass loss, i.e. T_95%_∼100 °C as reported in Table 3; but the heating curves of bark and sapwood condensed tannins showed strong similarity until the second onset of degradation located at T_d2≈_220 °C. Both displayed mass losses of 13.8% against 15% for heartwood condensed tannins (Fig. 7a); that trend of low thermal stability for the heartwood condensed tannins increased with temperature. Although the thermal curves at 80% residual mass supported the lesser thermal stability of heartwood condensed tannins, which degraded at T_80%_ = 275 °C; the thermal discrimination between the bark and sapwood increasing also for T > T_d2_ highlighted the low thermal stability of the bark condensed tannins. which degraded at T_80%_ = 362.5, against T_80%_ = 425 °C for the sapwood tannins (Fig. 7a). The better resistance of Okoume bark and sapwood condensed tannins to thermal degradation was corroborated by their residual rate at the end of decomposition process (T = 600 °C), with a residual mass of 78.5 and 78.9% for the bark and sapwood respectively, vs 73% for heartwood. This result showed a strong thermal stability of the three Okoume condensed tannins for temperatures up to 500 °C, which suggested a similar degree of polymerization as well as interflavonoid bonds in Okoume bark and sapwood condensed tannins. However, the close thermal stability between the bark and sapwood condensed tannins observed in this study suggested that the molecular structure and condensed tannins length of these two Okoume waste should not be very different. Furthermore, the similar thermal behavior between these two condensed tannins was supported by their DTG second step of degradation curves which displayed *T*_*max*_ = 244.99 for the bark and sapwood tannins (Fig. 7b). It was noteworthy that these *T*_*max*_ values were slightly higher than that found for quebracho condensed tannin, i.e. *T*_*max*_ = 240 °C^57^, against a value of *T*_*max*_ = 255.64 °C for Okoume, very close to that obtained for *Acacia dealbata*, at *T*_*max*_ = 255.77 °C^59^. Moreover, the good thermal stability of Okoume condensed tannins was also corroborated by its high value (T_d2_≈219.6 °C) compared to Aleppo pine tannins which started to degrade at 179 °C^57^, or other commercial tannins such as mimosa, quebracho and maritime pine which start to decompose at 146, 145 and 130 °C, respectively^60^.

**Figure 8:**
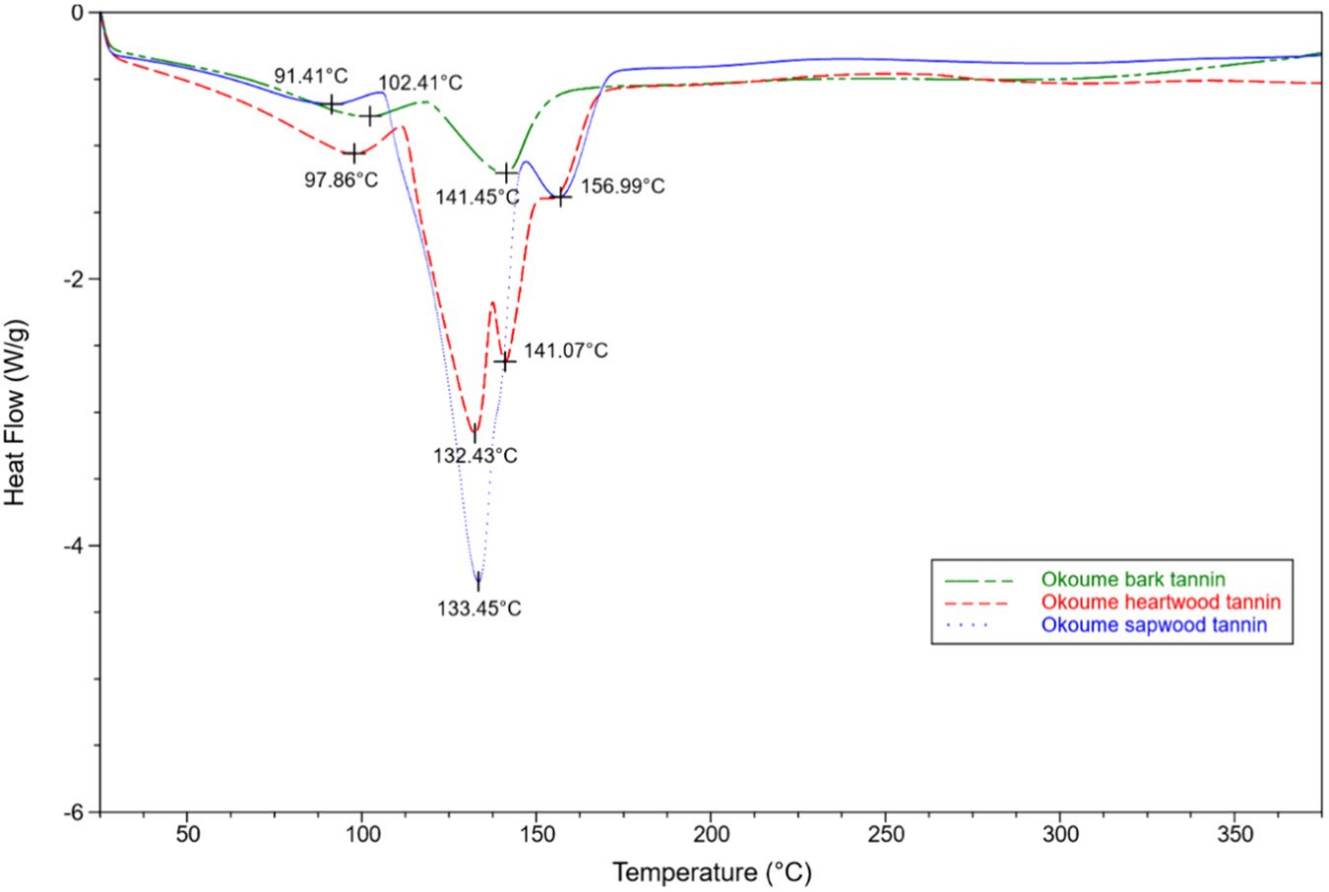
DSC of Okoume tannins heated at 10 °C/min in nitrogen (bark = green; sapwood tannins =  blue and heartwood tannins =  red).

The three Okoume condensed tannins were analyzed by DSC, which is the most widely accepted method for determining the glass transition temperature (T_g_) of natural or synthetic polymers^59^. That method was carried out at rate of 10 °C/min, and the thermograms were depicted in Fig. 8. All the condensed tannins showed typical endothermic peaks of elimination reactions^61,62^, and the first endotherm corresponded to changes in the heating curve at 102.4, 91.4, and 97.8 °C for the bark, sapwood and heartwood condensed tannins respectively. These peaks should be assigned to the loss of absorbed water as previously observed for other tannins^63,64^.

That loss of water was also noted in the strong peaks centered at 133.4 and 132.4 °C for the sapwood and heartwood condensed tannins (Fig. 8). However, additional endothermic peaks of water loss were observed at 141.4 and 141.07 °C for the bark and heartwood condensed tannins although this peak appeared as a shoulder near 141.07 °C for the sapwood tannins. However, the thermal curves of Fig. 8 revealed some thermal differences among the examined condensed tannins as shown by the peak at 156.9 °C which occurred only in the sapwood and heartwood condensed tannins. Although various studies attributed the four thermal areas of Fig. 8 to the loss of absorbed water in tannins, some authors have found that tannin monomers like catechin showed two endothermic features at 100 and 150 °C, due to the losses of associated water^57^ by this polyphenol. That result suggests a strong impact of the molecular structure on the thermal stability of condensed tannins. Thus, the differences observed in the thermal stability of Okoume condensed tannins should be connected not only to their fisetinidin, gallocatechin, catechin or robitinidin monomers content variability, but also to the condensation of these units in the polyphenol chains. With the exception of the dehydration temperature below 84.43 °C, all the Tg values of Okoume condensed tannins were above 104, 113, and 126 °C respectively, for chestnut, mimosa and quebracho tannins in previous works^59,63^. That finding emphasized the better thermal stability of Okoume condensed tannins.

## Conclusions

The study of Okoume extract was carried out to achieve three main objectives: (i) to quantify its total polyphenol content according to a colorimetric method; (ii) to characterize for the first time this bark’s condensed tannin extracts by MALDI-ToF, LC-MS and FTIR; and (iii) to understand the thermal behavior of Okoume condensed tannins. Maldi-ToF spectroscopy revealed the presence of distinctive compounds condensed tannins which contained a notable rate of hydrolysable tannins bearing galloyl moieties, and the presence of oligomers of glycosylated condensed tannins. The molecular structure of condensed tannins obtained by maceration of bark in an acetone/water mixture highlighted the presence of fisetinidin, gallocatechin and trihydroxyflavan as the major monomers. The highest oligomer chains detected in this condensed tannin were tetramers. A series of oligomers formed by a flavonoid trimer linked to up to ten sugar units was also identified for the first time. Stiasny number calculation and thermal analysis revealed the potential of Okoume wood wastes for use as raw material for condensed tannins with strong adhesive properties.

## Supplementary information

Supplementary information

## Data Availability

The data can be made available for sharing with third parties.

## References

[1] Yoan AO, Xue Y, Kiki MJM (2018). Gabon Wood Industry and Chinese Companies Activities. OALib.

[2] Nze Nguema, S. Présentation du Secteur Forestier au Gabon: Rapport sur l’évolution de la mise en oeuvre de la politique du Gouvernement dans les secteurs Forêts, Pêches et Aquatculture, Aires protégées et Formation. Rapport sur l’évolution de la mise en oeuvre de la politique du Gouvernement dans les secteurs Forêts, Pêches et Aquatculture, Aires protégées et Formation; Available from: http://www.euflegt.efi.int/documents/10180/23275/Pr%C3%A9sentation+du+Secteur+Forestier+au+Gabon/eb4427e5-f61c-4b09-83c3-6ab5bcfa14b4?version=1.0 (2009).

[3] Eyi Obame, A. P., Safou-Tchiama, R. & Kombila, M. Niveau de valorisation des déchets d’exploitation forestière: identification et estimation des rebuts de bois dans l’AAC 2014 de la SEEF a Nzamaligue. R*esearchGate [Internet]*, Available from: https://www.researchgate.net/publication/326092700_Niveau_de_valorisation_des_dechets_d%27exploitation_forestiere_identification_et_estimation_des_rebuts_de_bois_dans_l%27AAC_2014_de_la_SEEF_a_Nzamaligue (2017).

[4] Tessier AM, Delaveau P, Piffault N (1982). Oléo-Résine d’Aucoumea klaineana. Planta Med..

[5] Delaveau P, Vidal-Tessier AM (1988). Constituants secondaires à activité biologique du bois de quelques espèces tropicales. Bull. Société Bot. Fr. Actual. Bot.

[6] Delaveau P, Lallouette P, Tessier AM (1980). Drogues Végétales Stimulant l’Activité Phagocytaire du Système Réticuio-Endothélial1. Planta Med..

[7] Dongmo PMJ (2010). Chemical characterization, antiradical, antioxidant and anti-inflammatory potential of the essential oils of Canarium schweinfurthii and Aucoumea klaineana (Burseraceae) growing in Cameroon. Agric. Biol. J. N. Am..

[8] Renimel, I. & Andre, P. Use of an okume resin extract in the cosmetic and pharmaceutical fields, and in particular in the dermatological field. (2004).

[9] Rhourri‐Frih B (2009). Analysis of pentacyclic triterpenes by LC–MS. A comparative study between APCI and APPI. J. Mass. Spectrom..

[10] Bakraji EH, Salman N, Othman I (2002). Radiation-induced polymerization of acrylamide within Okoume (Aucoumea klaineana pierre). Radiat. Phys. Chem..

[11] Safou-Tchiama R, de Jéso B, Akagah AG, Sèbe G, Pétraud M (2007). A preliminary survey of the interfacial bonding of some tropical hardwoods towards succinic anhydride and 2-octen-1-yl succinic anhydride molecules: Impact of lignin and carbohydrate polymers structure on the chemical reactivity. Ind. Crop. Prod..

[12] Safou-Tchiama R, Barhé TA, Soulounganga P, Akagah AG, De Jeso B (2017). A comparative study of the syringyl, guaiacyl and hydroxyl groups units distribution in some African tropical hardwoods’ lignin by Py-GC/MS and spectroscopic techniques. J. ME S.

[13] Safou-Tchiama R, Obame SN, Brosse N, Soulounganga P, Barhé TA (2016). Investigating the potential of Aucoumea klaineana Pierre sapwood and heartwood wastes to produce cellulosic ethanol. Afr. J. Biotechnol..

[14] Mounguengui S (2016). Total phenolic and lignin contents, phytochemical screening, antioxidant and fungal inhibition properties of the heartwood extractives of ten Congo Basin tree species. Ann. For. Sci..

[15] Singleton VL, Rossi JA (1965). Colorimetry of total phenolics with phosphomolybdic-phosphotungstic acid reagents. Am. J. Enol. Vitic..

[16] Salmana M, Abdel-Hameeda E-SS, Bazaida SA, Al-Shamranib MG, Mohamedb HF (2014). Liquid chromatography-mass spectrometry (LC-MS) method for the determination of sugars in fresh pomegranate fruit juices. Der Pharma Chemica.

[17] Scalbert A, Monties B, Janin G (1989). Tannins in wood: comparison of different estimation methods. J. Agric. Food Chem..

[18] Broadhurst RB, Jones WT (1978). Analysis of condensed tannins using acidified vanillin. J. Sci. Food Agric..

[19] Voulgaridis E, Grigoriou A, Passialis C (1985). Investigations on bark extractives of Pinus halepensis Mill. Holz Als Roh- Werkst..

[20] Sanchez-De Melo I (2015). N-glycosylation profile analysis of Trastuzumab biosimilar candidates by Normal Phase Liquid Chromatography and MALDI-TOF MS approaches. J. Proteom..

[21] Stevanovic, T. & Perrin, D. Les extractibles du bois [Internet]. Presses Polytechniques et Universitaires Romandes, 242 p. Available from: /Sciences/Livre/chimie-du-bois-9782880747992 (2009).

[22] Chupin L, Motillon C, Charrier-El Bouhtoury F, Pizzi A, Charrier B (2013). Characterisation of maritime pine (*Pinus pinaster*) bark tannins extracted under different conditions by spectroscopic methods, FTIR and HPLC. Ind. Crop. Prod..

[23] Lucci P, Saurina J, Núñez O (2017). Trends in LC-MS and LC-HRMS analysis and characterization of polyphenols in food. TrAC. Trends Anal. Chem..

[24] Ahn JH (2018). Identification of antioxidant constituents of the aerial part of Plantago asiatica using LC–MS/MS coupled DPPH assay. Phytochem. Lett..

[25] Huang Y (2019). Antioxidant response of cucumber (Cucumis sativus) exposed to nano copper pesticide: Quantitative determination via LC-MS/MS. Food Chem..

[26] Ricci A (2017). Analytical profiling of food-grade extracts from grape (Vitis vinifera sp.) seeds and skins, green tea (Camellia sinensis) leaves and Limousin oak (Quercus robur) heartwood using MALDI-TOF-MS, ICP-MS and spectrophotometric methods. J. Food Compos. Anal..

[27] Galichet A, Sockalingum GD, Belarbi A, Manfait M (2001). FTIR spectroscopic analysis of Saccharomyces cerevisiae cell walls: study of an anomalous strain exhibiting a pink-colored cell phenotype. FEMS Microbiol. Lett..

[28] Kato K, Nitta M, Mizuno T (1973). Infrared spectroscopy of some mannans. Agric. Biol. Chem..

[29] Kacuráková M, Mathlouthi M (1996). FTIR and laser-Raman spectra of oligosaccharides in water: characterization of the glycosidic bond. Carbohydr. Res..

[30] Saad H (2012). Characterization of pomegranate peels tannin extractives. Ind. Crop. Prod..

[31] Bikoro Bi Athomo A (2018). Chemical composition of African mahogany (*K. ivorensis* A. Chev) extractive and tannin structures of the bark by MALDI-TOF. Ind. Crop. Prod..

[32] Callemien D, Collin S (2008). Use of RP-HPLC-ESI (–)-MS/MS to differentiate various proanthocyanidin isomers in lager beer extracts. J. Am. Soc. Brew. Chem. J Am Soc Brew Chem.

[33] Yasir M, Sultana B, Amicucci M (2016). Biological activities of phenolic compounds extracted from Amaranthaceae plants and their LC/ESI-MS/MS profiling. J. Funct. Foods.

[34] Awolola GV, Koorbanally NA, Chenia H, Shode FO, Baijnath H (2014). Antibacterial and Anti-Biofilm Activity of Flavonoids and Triterpenes Isolated from The Extracts of Ficus Sansibarica Warb. Subsp. Sansibarica (Moraceae) Extracts. Afr. J. Tradit. Complement. Altern. Med..

[35] Grasel F, dos S, Ferrão MF, Wolf CR (2016). Development of methodology for identification the nature of the polyphenolic extracts by FTIR associated with multivariate analysis. Spectrochim. Acta A Mol. Biomol. Spectrosc..

[36] Ricci A (2016). Spectroscopy analysis of phenolic and sugar patterns in a food grade chestnut tannin. Food Chem..

[37] Ucar MB, Ucar G, Pizzi A, Gonultas O (2013). Characterization of Pinus brutia bark tannin by MALDI-TOF MS and 13C NMR. Ind. Crop. Prod..

[38] Jaitz L (2010). LC–MS/MS analysis of phenols for classification of red wine according to geographic origin, grape variety and vintage. Food Chem..

[39] Bursal E, Köksal E, Gülçin İ, Bilsel G, Gören AC (2013). Antioxidant activity and polyphenol content of cherry stem (Cerasus avium L.) determined by LC–MS/MS. Food Res. Int..

[40] Quifer-Rada P (2015). A comprehensive characterisation of beer polyphenols by high resolution mass spectrometry (LC–ESI-LTQ-Orbitrap-MS). Food Chem..

[41] Falcão L, Araújo MEM (2013). Tannins characterization in historic leathers by complementary analytical techniques ATR-FTIR, UV-Vis and chemical tests. J. Cult. Herit..

[42] Grasel, F., dos, S., Ferrão, M. F., Wolf, C. R. & Angélica, R. Characterization of Natural Tanning Extracts by FTIR and Multivariate Analysis. *XXXIII IULTCS Congress* (2015).

[43] Bianchi S, Gloess AN, Kroslakova I, Mayer I, Pichelin F (2014). Analysis of the structure of condensed tannins in water extracts from bark tissues of Norway spruce (Picea abies [Karst.]) and Silver fir (Abies alba [Mill.]) using MALDI-TOF mass spectrometry. Ind. Crop. Prod..

[44] Blanchon JA, Mabiala J-ND (1993). référentiel et générique en Kiyoombi (H 12b): étude synchronique. Lab. Phon. Linguist. Afr. CRLS-Univ Lumière-Lyon.

[45] Bouet C (1980). La saga de l’okoumé au Gabon. Cah. ORSTOM Sér. Sci. Hum..

[46] Davis BD, Brodbelt JS (2004). Determination of the glycosylation site of flavonoid monoglucosides by metal complexation and tandem mass spectrometry. J. Am. Soc. Mass. Spectrom..

[47] Kachlicki P, Einhorn J, Muth D, Kerhoas L, Stobiecki M (2008). Evaluation of glycosylation and malonylation patterns in flavonoid glycosides during LC/MS/MS metabolite profiling. J. Mass. Spectrom..

[48] Drovou S (2015). Flavonoid tannins linked to long carbohydrate chains–MALDI-TOF analysis of the tannin extract of the African locust bean shells. Ind. Crop. Prod..

[49] Pizzi A, Cameron FA (1986). Flavonoid tannins: structural wood components for drought-resistance mechanisms of plants. Wood Sci. Technol. USA [Internet]..

[50] Boeriu CG, Bravo D, Gosselink RJA, van Dam JEG (2004). Characterisation of structure-dependent functional properties of lignin with infrared spectroscopy. Ind. Crop. Prod..

[51] Geethu M, Suchithra P, Kavitha C, Aswathy J, Dinesh Babu K (2014). Murugan. Fourier-transform infrared spectroscopy analysis of different solvent extracts of water hyacinth (eichhornia crassipes mart solms.). An. allelopathic approach -. World J. Pharm. Pharm Sci..

[52] Ping L, Brosse N, Chrusciel L, Navarrete P, Pizzi A (2011). Extraction of condensed tannins from grape pomace for use as wood adhesives. Ind. Crop. Prod..

[53] Yazaki Y, Collins PJ (1994). Wood adhesives from Pinus radiata bark. Holz Als Roh- Werkst..

[54] Navarrete P (2012). Low. Formaldehyde Emitting Biobased Wood Adhesives Manufactured Mixtures Tannin Glyoxylated Lignin.

[55] Sekaran G, Thamizharasi S, Ramasami T (2001). Physicochemical and thermal properties of phenol–formaldehyde-modified polyphenol impregnate. J. Appl. Polym. Sci..

[56] Baaka N, Ammar M, Saad MK, Khiari R (2017). Properties of Tannin-Glyoxal Resins Prepared from Lyophilized and Condensed Tannin. J. Text. Eng. Fash. Technol..

[57] Gaugler M, Grigsby WJ (2009). Thermal Degradation of Condensed Tannins from Radiata Pine Bark. J. Wood Chem. Technol..

[58] Zhao Z, Umemura K (2015). Investigation of a New Natural Particleboard Adhesive Composed of Tannin and Sucrose. 2. Effect of Pressing Temperature and Time on Board Properties, and Characterization of Adhesive. Bioresour..

[59] Lisperguer, J., Saravia, Y. & Vergara, E. Structure and thermal behavior of tannins from acacia dealbata bark and their reactivity toward formaldehyde. *J. Chil. Chem. Soc*. **61**(4), 3188–90 (2016).

[60] Saad H, Khoukh A, Ayed N, Charrier B, Bouhtoury FC-E (2014). Characterization of Tunisian Aleppo pine tannins for a potential use in wood adhesive formulation. Ind. Crop. Prod..

[61] Shnawa HA, Khalaf MN, Jahani Y, Taobi AAH (2015). Efficient thermal stabilization of polyvinyl chloride with Tannin-Ca Complex as Bio-Based Thermal Stabilizer. Mater. Sci. Appl..

[62] Shnawa HA, Jahani Y, Khalaf MN, Taobi AH (2016). The potential of tannins as thermal co-stabilizer additive for polyvinyl chloride. J. Therm. Anal. Calorim..

[63] Carsote, C. *et al*. Effect of temperature and relative humidity on vegetable tanned leather studied by thermal analysis. In: *ICAMS—5th International Conference on Advanced Materials and Systems*, pp 505–510 (2014).

[64] Ying, G. *et al*. Testing of artificially aged leather in acid rain. In*: the 5th International Conference on Advanced Materials and Systems*, p 567 (2014).

