# Supplementary material for: The condensed tannins of Okoume (Aucoumea klaineana Pierre): A molecular structure and thermal stability study
Source: Sci Rep. 2020 Feb 4;10:1773. doi: 10.1038/s41598-020-58431-7 (PMC7000823; doi:10.1038/s41598-020-58431-7)
Supplement: Supplementary file 1 — Supplementary information [file 41598_2020_58431_MOESM1_ESM.docx]

**Supplementary Information**

**The condensed tannins of Okoume (*Aucoumea klaineana* Pierre): A molecular structure and thermal stability study**

**Starlin Péguy Engozogho Anris^1,2*^, Arsène Bikoro Bi Athomo^1,2^, Rodrigue Safou Tchiama^1,3^, Francisco José Santiago-Medina^4^, Thomas Cabaret^1^, Antonio Pizzi^4^, and Bertrand Charrier^1^**

^1^CNRS/Université de Pau des Pays de l’Adour, Institut des sciences analytiques et de physico-chimie pour l’environnement et les matériaux, Xylomat, UMR5254, 40004, Mont de Marsan, France

^2^ Laboratoire de Recherche et de Valorisation du Matériau Bois (LaReVa Bois). Ecole Normale Supérieure d’Enseignement Technique (ENSET). BP 3989, Libreville (Gabon).

^3^ Laboratoire des Substances Naturelles et de Synthèse Organométalliques (LASNSOM). Unité de Recherche en Chimie, Université des Sciences et Techniques de Masuku. BP.941, Franceville (Gabon).

^4^ EINSTIB-LERMAB, Université de Lorraine. 27 rue Philippe Seguin, BP 1041, 88051 Epinal, France

**Supplementary Table 1**. Molecular structure as derived by Maldi-ToF of condensed tannins extracted in

Okoume bark by acetone/water.

| **Designation** | **m/z (Da)** | **Label** | **Molecular structure** |
| --- | --- | --- | --- |
| Benzoate acid | 121 | I |  |
| p-Hydroxybenzoate acid | 137.12 | H |  |
| Gallic acid | 166 | E |  |
| Glycosyl unit | 180 | Gly |  |
| Dihydroxyflan- 2*x*H | 240,27 | P’ |  |
| Dihydroxyflan | 242.27 | P |  |
| Trihydroxyflavan – 2xH | 255.6 | F’ |  |
| Trihydroxyflavan | 257.6 | F |  |
| Fisetinidin | 274.4 | A |  |
| Catechin-3*x*H | 287.4 | K |  |
| Epicatechin | 290.27 | Q |  |
| Gallocatechin – 2xH | 303 | D’ |  |
| Gallocatechin | 305.5 | D |  |
| Cellobiose – 2*x*H | 340.2 | Gly_2_ |  |
| Dihydroxyflavan-3-p-hydroxybenzoate  or  Trihydroxyflavan-3-benzoate | 362.1 | G_1_H_1_ or F_1_I_1_ |  |
| Dihydroxyflavan-glycosyl type unit | 405.8 | P_1_Gly_1_ |  |
| Fisetinidin-glycosyl type unit | 438.0 | A_1_Gly_1_ |  |
| (-)-epicatechin-3-gallate | 438.0 | Q_1_E_1_ |  |
| Fisetinidin-glycosyl type unit | 452 | A_1_Gly_1_ |  |
| Dihydroxyflan | 481.6 | P_2_ |  |
| Trihydroxyflavan-Fisetinidine | 533.6 | F_1_A_1_ |  |
| Fisetinidin | 539.6 | A_2_ |  |
| Fisetinidin-gallocatechin | 575.2 | A_1_D_1_ |  |
| Isoquercetingallate | 617.4 | R_1_E_1_ |  |
| Fisetinidin-gallocatechin | 850.7 | A_2_D_1_ |  |
| Fisetinidin-gallocatechin | 856.8 | A_2_D |  |
| Fisetinidin-gallocatechin-glycosyl type unit | 1012.6 | A_2_DGly_1_ |  |
| Fisetinidin-catechin | 1128.3 | A_2_C_2_ |  |
| Fisetinidin-gallocatechin-Glycosyl type unit | 1174.4 | A_2_DGly_2_ |  |
| Fisetinidin-Gallocatechin-Glucosyl type unit | 1336.2 | A_2_DGly_3_ |  |
| Fisetinidin-Gallocatechin- Glucosyl type unit | 1498.1 | A_2_DGly_4_ |  |
| Fisetinidin-Gallocatechin- Glucosyl type unit | 1660.1 | A_2_DGly_5_ |  |
| Fisetinidin-Gallocatechin- Glucosyl type unit | 1822.2 | A_2_DGly_6_ |  |
| Fisetinidin-Gallocatechin-glycosyl type unit | 1984.4 | A_2_DGly_7_ |  |
| Fisetinidin-Gallocatechin- Glucosyl type unit | 2146.6 | A_2_DGly_8_ |  |
| Fisetinidin-Gallocatechin- Glucosyl type unit | 2309.0 | A_2_DGly_9_ |  |
| Fisetinidin-Gallocatechin- Glucosyl type unit | 2471.0 | A_2_DGly_10_ |  |
